# Pathogenesis and Transmissibility of North American Highly Pathogenic Avian Influenza A(H5N1) Virus in Ferrets

**DOI:** 10.3201/eid2809.220879

**Published:** 2022-09

**Authors:** Joanna A. Pulit-Penaloza, Jessica A. Belser, Nicole Brock, Poulami Basu Thakur, Terrence M. Tumpey, Taronna R. Maines

**Affiliations:** Author affiliation: Centers for Disease Control and Prevention, Atlanta, Georgia, USA

**Keywords:** influenza, avian influenza, ferret, transmission, cell culture, viruses, respiratory infections, H5N1, zoonoses, vaccine-preventable diseases

## Abstract

Highly pathogenic avian influenza A(H5N1) viruses have spread rapidly throughout North American flyways in recent months, affecting wild birds in over 40 states. We evaluated the pathogenicity and transmissibility of a representative virus using a ferret model and examined replication kinetics of this virus in human respiratory tract cells.

Highly pathogenic avian influenza (HPAI) A(H5Nx) viruses (clade 2.3.4.4, primarily H5N2 and H5N8 subtypes) were first detected along the Pacific flyway in 2014, resulting in outbreaks in wild bird and domestic poultry populations in North America (*1*). No human cases were associated with these outbreaks in the United States, but sporadic HPAI H5Nx virus human infections have been documented in other geographic locations, highlighting the potential of these viruses to jump species barriers during culling or sampling of infected birds (*2*). Despite reduced detection of H5Nx viruses in North America in recent years, clade 2.3.4.4b H5N1 virus, which emerged and displaced other H5Nx virus in Europe, Asia, and Africa, was detected in wild birds in North America in late 2021. Since then, the virus has been introduced into all 4 flyways of North America (*3*). The detection and spread of this virus in US commercial and backyard poultry pose substantial economic implications and concerns for human health, as evidenced by the first confirmed HPAI H5N1 human case, documented in the United States in April 2022 (*4*), underscoring the pandemic potential presented by continued circulation of viruses at the animal–human interface. To investigate the relative risk posed by these viruses, we examined the pathogenicity and transmissibility of a representative HPAI H5N1 virus, A/American Wigeon/SC/22-000345-001/2021 (aw/SC) by using a ferret model and assessed the capacity of this virus to replicate in a human respiratory cell line compared with seasonal H1N1 viruses. 

We inoculated 6 ferrets with 6 log_10_ 50% egg infectious dose (EID_50_) of aw/SC virus (GISAID accession no. EPI_ISL_9869760; https://www.gisaid.org) and observed them for 9 days. The animals became productively infected, but the disease was generally mild; ferrets exhibited <5% weight loss and transient fever (elevated by 1.1°C) (Table). We observed no sneezing or nasal discharge during infection. Virus replication in tissues collected on day 3 postinoculation from 3 additional ferrets was limited to the upper respiratory tract except for 1 animal that had low-level virus in the lungs. We did not detect infectious virus in blood, intestines, olfactory bulb, brain, kidneys, spleen, or liver (data not shown). That finding indicates less extensive tissue dissemination of the aw/SC virus compared with previously evaluated clade 2.3.4.4 North America H5Nx viruses, which we observed throughout the ferret respiratory tract and in some olfactory bulb and intestinal samples (*5*).

**Table:** Results of ferrets inoculated with influenza A(H5N1) virus isolate A/American Wigeon/SC/22–000345–001/2021*

Parameter	Value
Weight loss, %†	4.7
Temperature increase, °C‡	1.1
Nasal wash titer (peak titer days)§	4.6 (1–5)
Virus titer at day 3 postinoculation¶	
Nasal wash	2.4 (3/3)
Nasal turbinates	3.5 (3/3)
Soft palate	2.9 (3/3)
Trachea	ND
Lung	2.2 (1/3)

*Six ferrets were inoculated with 6 log_10_ EID_50_ of A/American Wigeon/SC/22-000345-001/2021 (GISAID identical virus sequence accession no. EPI_ISL_9869760, https://www.gisaid.org; in a 1-mL volume. Three additional ferrets were inoculated and then euthanized and tested for virus titer at day 3 postinoculation. EID_50_, 50% egg infectious dose; ND, not detected †Percentage mean maximum weight loss within 9 days postinoculation. ‡Mean maximum rise in body temperature from baseline (37.6°C–39.4°C). §Mean maximum virus titer in nasal washes, expressed as log_10_ EID_50_/mL. ¶Viral titers are expressed as log_10_ EID_50_/mL, except for lung titer, which is expressed as log_10_ EID_50_/g of tissue. Number of ferrets with detectable virus is specified in parentheses. Limit of virus detection was 1.5 log_10_ EID_50_/mL or g.

Next, we evaluated transmissibility of the aw/SC H5N1 virus. We detected infectious virus in nasal wash specimens among all inoculated ferrets up to day 7 postinoculation (Figure, panels A, B). Mean peak nasal wash titers (4.6 log_10_ EID_50_/mL) were comparable to those observed for the 2014 North America H5Nx isolates that did not transmit between ferrets (<4.2 log_10_ EID_50_/mL) (*5*). However, the aw/SC virus did not transmit in a direct-contact setting (cohoused inoculated and naive ferret pairs) or through the air (ferret pairs in separate cages with perforated cage walls), as evidenced by lack of virus detection in nasal washes and lack of seroconversion of the contact ferrets to homologous virus.

**Figure:**
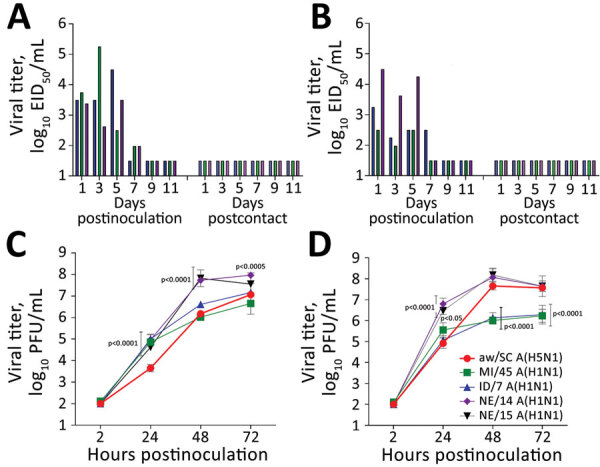
Evaluating influenza A(H5N1) virus isolate aw/SC using in vivo and in vitro models. A, B) Ferrets were intranasally inoculated with 6 log_10_ EID_50_ of aw/SC virus in 1 mL of phosphate-buffered saline and direct contact (A) and respiratory droplet (B) transmission models were established with naive ferrets (1:1 ratio) the following day as described previously (*9*). Nasal wash samples were collected from inoculated and contact ferrets every other day, and virus titers were determined in eggs (*10*). As shown, infectious virus was detected in nasal wash specimens from all inoculated ferrets up to day 7 (left side of each panel); however, ferrets exposed only by direct contact (panel A, right) or through the air (panel B, right) did not show infectious virus. C, D) Replication kinetics of aw/SC virus were evaluated in human respiratory tract cells and compared with the H1N1 viruses, A/Michigan/45/2015 (MI/45), A/Idaho/7/2018 (ID/7), A/Nebraska/14/2019 (NE/14), and A/Nebraska/15/2018 (NE/15). Calu-3 cells (ATCC, https://www.atcc.org) were grown to confluence under submerged conditions in 12-mm diameter transwell inserts (Corning, https://www.corning.com). The cells were infected apically at a multiplicity of infection of 0.01 for 1 h and then washed and incubated at 33°C (C) or 37°C (D) as previously described (*6*). Virus titers in triplicate cell-supernatant samples were determined by standard plaque assay in MDCK cells (*10*). The limit of virus detection was 1.5 log_10_ EID_50_/mL or 2 log_10_ PFU/mL. Error bars indicate SDs. p values provided for avian H5N1 versus human seasonal H1N1 viruses were calculated by 2-way analysis of variance with a Tukey posttest. aw/SC, A/American Wigeon/SC/22-000345-001/2021; EID_50_, 50% egg infectious dose.

Prior evaluations of North America HPAI H5N2 and H5N8 isolates in human airway cells demonstrated that these viruses were capable of productive replication, albeit at reduced titers compared with virulent H5N1 and seasonal H1N1 viruses (*5*). To assess whether human bronchial epithelial cells support replication of the aw/SC H5N1 virus, we compared growth kinetics of this virus with contemporary H1N1 strains at 33°C and 37°C in Calu-3 cells (ATCC, https://www.atcc.org), a cell line that permits concurrent evaluation of human and zoonotic influenza viruses for risk assessment evaluations (Figure, panels C, D) (*6*). At 37°C, aw/SC H5N1 virus reached comparable peak mean titer to all H1N1 viruses tested by 48 hours postinoculation. However, at 33°C, aw/SC showed a substantial delay in virus replication at 24 hours postinoculation compared with all tested H1N1 strains (p<0.0001). This delay could, in part, be explained by the lack of E627K and D701N substitutions in the polymerase basic protein 2 sequence of the aw/SC virus, substitutions that are considered critical for the mammalian adaptation of avian influenza viruses (*7*). Although we noted strain-specific differences between all viruses, the data indicate that aw/SC virus can replicate efficiently in the types of cells that woke up the human respiratory tract.

The introduction of HPAI H5N1 viruses into multiple North America flyways represents a substantial concern for human health. Continued circulation of viruses in wild bird populations and repeated introduction of these viruses into gallinaceous poultry confer a multitude of opportunities for these viruses to acquire molecular features associated with enhanced mammalian fitness and human infection. Our data support the Influenza Risk Assessment Tool determination that HPAI H5N1 viruses do not pose a substantial risk to public health at this time (*8*). However, close surveillance of circulating strains and continued assessment of new viruses are crucial to ensure strains with enhanced mammalian fitness are quickly identified.

## References

[1] Lee DH, Torchetti MK, Winker K, Ip HS, Song CS, Swayne DE. Intercontinental spread of Asian-origin H5N8 to North America through Beringia by migratory birds. J Virol. 2015;89:6521–4. 10.1128/JVI.00728-15 25855748 PMC4474297

[2] Wille M, Barr IG. Resurgence of avian influenza virus. Science. 2022;376:459–60. 10.1126/science.abo1232 35471045

[3] Bevins SN, Shriner SA, Cumbee JC Jr, Dilione KE, Douglass KE, Ellis JW, et al. Intercontinental movement of highly pathogenic avian influenza A(H5N1) clade 2.3.4.4 virus to the United States, 2021. Emerg Infect Dis. 2022;28:1006–11. 10.3201/eid2805.220318 35302933 PMC9045435

[4] Centers for Disease Control and Prevention. US case of human avian influenza A(H5) virus reported. 2022 Apr 28 [cited 2022 Apr 28]. https://www.cdc.gov/media/releases/2022/s0428-avian-flu.html

[5] Pulit-Penaloza JA, Sun X, Creager HM, Zeng H, Belser JA, Maines TR, et al. Pathogenesis and transmission of novel highly pathogenic avian influenza H5N2 and H5N8 viruses in ferrets and mice. J Virol. 2015;89:10286–93. 10.1128/JVI.01438-15 26223637 PMC4580194

[6] Zeng H, Goldsmith C, Thawatsupha P, Chittaganpitch M, Waicharoen S, Zaki S, et al. Highly pathogenic avian influenza H5N1 viruses elicit an attenuated type i interferon response in polarized human bronchial epithelial cells. J Virol. 2007;81:12439–49. 10.1128/JVI.01134-07 17855549 PMC2169033

[7] Nuñez IA, Ross TM. A review of H5Nx avian influenza viruses. Ther Adv Vaccines Immunother. 2019;7:2515135518821625. 10.1177/2515135518821625 30834359 PMC6391539

[8] Centers for Disease Control and Prevention. Summary of Influenza Risk Assessment Tool (IRAT). H5N1 clade 2.3.4.4b [A/American wigeon/South Carolina/AH0195145/2021] [cited 2022 Mar 31]. https://www.cdc.gov/flu/pandemic-resources/monitoring/irat-virus-summaries.htm#H5N122

[9] Maines TR, Chen LM, Matsuoka Y, Chen H, Rowe T, Ortin J, et al. Lack of transmission of H5N1 avian-human reassortant influenza viruses in a ferret model. Proc Natl Acad Sci U S A. 2006;103:12121–6. 10.1073/pnas.0605134103 16880383 PMC1567706

[10] Szretter KJ, Balish AL, Katz JM. Influenza: propagation, quantification, and storage. Curr Protoc Microbiol. 2006;15:15G.1.1–1.22. 10.1002/0471729256.mc15g01s3 18770580

